# All-depth dispersion cancellation in spectral domain optical coherence tomography using numerical intensity correlations

**DOI:** 10.1038/s41598-018-27388-z

**Published:** 2018-06-15

**Authors:** Mikkel Jensen, Niels Møller Israelsen, Michael Maria, Thomas Feuchter, Adrian Podoleanu, Ole Bang

**Affiliations:** 1 Technical University of Denmark, DTU Fotonik, Kongens Lyngby, 2800 Denmark; 2 NKT Photonics, Birkerød, 3460 Denmark; 3 University of Kent, School of Physical Sciences, Canterbury, Kent CT2 7NZ England

## Abstract

In ultra-high resolution (UHR-) optical coherence tomography (OCT) group velocity dispersion (GVD) must be corrected for in order to approach the theoretical resolution limit. One approach promises not only compensation, but complete annihilation of even order dispersion effects, and that at all sample depths. This approach has hitherto been demonstrated with an experimentally demanding ‘balanced detection’ configuration based on using two detectors. We demonstrate intensity correlation (IC) OCT using a conventional spectral domain (SD) UHR-OCT system with a single detector. IC-SD-OCT configurations exhibit cross term ghost images and a reduced axial range, half of that of conventional SD-OCT. We demonstrate that both shortcomings can be removed by applying a generic artefact reduction algorithm and using analytic interferograms. We show the superiority of IC-SD-OCT compared to conventional SD-OCT by showing how IC-SD-OCT is able to image spatial structures behind a strongly dispersive silicon wafer. Finally, we question the resolution enhancement of $\sqrt{2}$ that IC-SD-OCT is often believed to have compared to SD-OCT. We show that this is simply the effect of squaring the reflectivity profile as a natural result of processing the product of two intensity spectra instead of a single spectrum.

## Introduction

In-depth imaging of human tissue has been one of the greatest achievements of optical technologies. Optical coherence tomography (OCT) was initiated more than 25 years ago, when a cross-sectional image of the human retina using a Michelson interferometer was demonstrated^1^. The ability to display changes of the refractive index by detecting photons balistically backscattered millimetres inside tissue at the micrometre scale has let to a revolution in the field of ophthalmology and is essential for many other medical fields^2^. Quite recently OCT has even been demonstrated for macroscopic imaging, thereby adding a new perspective in terms of its applications^3^.

With a Gaussian spectral profile the axial (in-depth) resolution limit is intrinsically given by $\delta z=\frac{2\,\mathrm{ln}\,2}{\pi }\frac{{\lambda }_{c}^{2}}{{\rm{\Delta }}\lambda }$, where, *λ*_*c*_ is the central wavelength and Δ*l* is the full-width at half maximum (FWHM) spectral bandwidth of the light source^2^. To maximize the penetration depth in tissue *λ*_*c*_ is typically chosen to be in the near infrared (NIR) regime to minimize scattering^2^, but well below the major absorption bands of water peaking at *λ* = 3 *μ*m^4^. In order to maintain *δz* when increasing the wavelength from the visible to the NIR, one is left to maximize Δ*λ*. In doing so, chromatic dispersion in both optical components and sample will degrade the depth resolution. This is due to each wavelength experiencing a different optical path through the system and sample, causing the optical path difference to differ as well. The effect of the different optical path lengths in the two paths (reference arm and sample arm) for different wavelengths is commonly known as the dispersion mismatch^2,5^.

To counter the dispersion mismatch, dispersion compensation (DC) is done hardware wise by ensuring that the two arms are constructed identically. However, this makes the set-up more costly and increases complexity. Instead simple DC with a glass plate, such as BK7, is today used to balance the dispersion^5^. Alternatively, a large variety of numerical approaches have been introduced, first for time domain (TD) OCT^6–8^ and later for spectral domain OCT (SD-OCT). In particular, in SD-OCT several new DC methods have been demonstrated^9–14^ that can achieve single-interface DC, i.e., sharpening only one interface in the sample. Only a few methods promise multi-interface DC, which is necessary in order to maintain axial resolution throughout the imaging depth of a multi-layered dispersive sample^15–22^. One approach that can compensate only second order dispersion at multiple interfaces, is the fractional Fourier transform combined with numerical segmentation of the sample and a radon transform, posing a heavy computational load, which scales with the number of pre-defined sample segmentations in depth^15^. Another simpler approach is to perform a linear interpolation of the depth-dependent DC from two depths where the dispersion mismatch is known^16^. Two alternative approaches, inspired by quantum OCT^17,18^, are phase conjugate OCT^19,20^ and chirped-pulse interferometry OCT^21,22^. These approaches can do even-order dispersion cancellation, but are costly and complex hardware-wise, due to the requirement of sum frequency generation, while providing only low sensitivity. A numerical scheme exploiting a generalized auto-convolution function for depth-dependent dispersion cancellation, also developed in the foot steps of quantum OCT, was proposed by Banaszek *et al*. and termed ‘blind dispersion compensation’^23^. This method promises protection from GVD using a conventional SD-OCT system with a single detector. Hardware implementations of Banaszek’s approach have also been proposed and termed ‘spectral intensity’ or ‘intensity-interferometric’ OCT^24–27^, but these again require two detectors and added complexity of the experimental set-up. We here consider the numerical technique of Banaszek and term it intensity correlation spectral domain OCT (IC-SD-OCT). All reports so far on implementing IC-SD-OCT, both numerical and hardware-wise, share two major drawbacks compared to conventional SD-OCT: (1) Halving of the imaging depth, and (2) the appearance of IC artefacts stemming from intensity cross terms. Extending the numerical scheme of Shirai *et al*.^28^, we here for the first time demonstrate ultra-high resolution SD-OCT with all-depth multi-interface sample dispersion removal with significant artefact reduction and full imaging depth using a conventional SD-OCT setup. We do this by numerically implementing the IC scheme, but on the analytical signal, which we distinguish from the standard IC scheme by denoting it ICA. An artefact reduction scheme similar to what is presented in^28^ is then applied to the ICA signal, and we show that the scheme works equally well using data from a conventional OCT setup. By imaging two different silicon phantoms, we highlight the applicability of IC-SD-OCT with a conventional SD-OCT set-up, and show that GVD is intrinsically removed at all depths of the sample with no depth segmentation or conventional DC needed, while maintaining the imaging depth.

## Theory

In this section the theory behind IC-SD-OCT is presented. First, we introduce the basic concept of IC-SD-OCT in the setting of conventional OCT, and we later apply the analytic signal to explain the image depth-maintaining procedure. Subsequently, the full mathematical framework of ICA-SD-OCT is presented, including the artefact reduction technique. Finally we discuss the axial resolution in IC-SD-OCT, and show numerical simulations to validate the theoretical predictions, and ICA-SD-OCT is compared to quantum OCT.

### Intensity correlation spectral-domain optical coherence tomography - IC-SD-OCT

**Figure 1:**
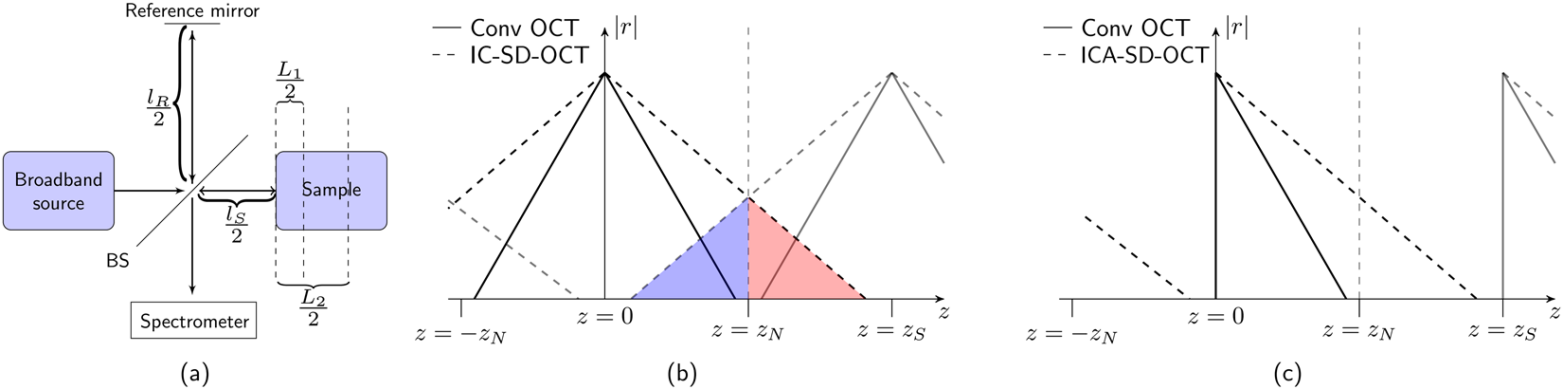
(**a**) Schematic drawing of the Michelson interferometer with path lengths employed in the text. BS is a beam splitter. (**b**) Schematic illustration of an A-scan before (solid) and after (dashed) the IC-SD-OCT procedure and (**c**) the ICA-SD-OCT procedure. The grey lines with a peak at *z* = *z*_*S*_ are mirror Fourier components from the discrete sampling of the spectrum. When the mirror components overlap on the real A-scan, aliasing occurs, which causes loss of information from the deep layers [red in (**b**)], as well as obscuration of the otherwise still visible part of the A-scan [blue in (**b**)]. In (**c**) the mirror terms do not interfere up to *z* = *z*_*S*_, and the full range of points can be used, such that the imaging depth is maintained through the ICA-SD-OCT procedure as opposed to the IC-SD-OCT procedure.

In SD-OCT, the channelled spectrum (interferogram) is given by

1 $$I(\omega )\propto {|{E}_{R}+{E}_{S}|}^{2}={|{E}_{R}|}^{2}+{|{E}_{S}|}^{2}+{E}_{R}{E}_{S}^{\ast }+{E}_{R}^{\ast }{E}_{S},$$

where *E*_*R*_ and *E*_*S*_ are the electric fields returned to the spectrometer from the reference arm and sample arm, respectively, see Fig. 1(a) for a sketch of the set-up. The electric field from the reference arm is

2 $${E}_{R}(\omega )=\sqrt{\frac{{I}_{0}(\omega )}{2}}{e}^{i\omega t-ik{l}_{R}},$$

and for two scattering centres in the sample arm, the electrical field from the sample can be written as

3 $${E}_{S}(\omega )=\sqrt{\frac{{I}_{0}(\omega )}{2}}{e}^{i\omega t-ik{l}_{S}}[{r}_{1}{e}^{-i\beta (\omega ){L}_{1}}+{r}_{2}{e}^{-i\beta (\omega ){L}_{2}}],$$

where *I*_0_ is the source spectrum, *r*_1_, *r*_2_ are the complex reflection coefficients, *l*_*s*_, *l*_*r*_ are the sample and reference paths, measured as twice the distance from the beam splitter to the sample surface and reference mirror, respectively. *L*_1_, *L*_2_ are twice the distances from the sample’s surface to each of the scattering centres, and $\beta (\omega )=\frac{\omega }{c}n(\omega )$ is the wavenumber in the sample, with *c* being the vacuum speed of light, and *n* being the depth-averaged refractive index of the sample. In general the depth-averaged refractive index will of course be different for two scattering centres at different depths, but we assume this difference to be negligible, such that *n*_1_(*ω*) ≈ *n*_2_(*ω*) ≡ *n*(*ω*).

Assuming real reflection coefficients, Eqs (1–3) and are combined and the normalised interferogram, *I*_*n*_ is obtained through $\,{I}_{n}=\frac{2I(\omega )-{I}_{0}(\omega )}{{I}_{0}(\omega )}$, yielding

4 $$\begin{array}{rcl}{I}_{n} & = & \frac{2I(\omega )-{I}_{0}(\omega )}{{I}_{0}(\omega )}={r}_{1}^{2}+{r}_{2}^{2}+2{r}_{1}{r}_{2}\,\cos \,({\rm{\Delta }}L\beta (\omega ))+2{r}_{1}\,\cos \,(\frac{\omega {\rm{\Delta }}l}{c}+\beta (\omega ){L}_{1})\\  &  & +\,2{r}_{2}\,\cos \,(\frac{\omega {\rm{\Delta }}l}{c}+\beta (\omega ){L}_{2})\end{array}$$

where Δ*L* = *L*_2_ − *L*_1_ and Δ*l* = *l*_*s*_ − *l*_*r*_. To generate the IC-SD-OCT interferogram, *I*_*n*_ is multiplied by itself, however flipped around the central frequency, *ω*_0_, and complex conjugated:

5 $${I}_{IC}({\omega }_{0},\omega {\rm{^{\prime} }})={I}_{n}({\omega }_{0}+\omega {\rm{^{\prime} }})\,{I}_{n}^{\ast }({\omega }_{0}-\omega {\rm{^{\prime} }})$$

where *ω*′ = *ω* − *ω*_0_. This intra-spectral product between the two optical frequency components ω_0_ + ω′ and ω_0_ − ω′ can be understood as probing the sample and the reference object at two different frequencies and seeking cross correlations between all the four electric fields involved, hence fourth-order field correlations. This classical approach is inspired by quantum OCT directly measuring fourth order correlations, which will be discussed in a later section. It is important to note that as IC-SD-OCT is a classical analogy to quantum OCT, the first realizations supposedly required two spectrometers to mimic the two photo detectors of quantum OCT, but Shirai showed that it is fundamentally equivalent to using two identical spectra obtained by one spectrometer instead of two different spectrometers in a ‘balanced detection’ configuration^29^. This means that *I*_*n*_(*ω*_0_ − *ω*′) in eq. (5) can be obtained either experimentally or numerically from *I*_*n*_(*ω*_0_ + *ω*′).

Equation (5) contains multiplication of cosines from eq. (4), which creates oscillations with half the initial period. As a result, all peaks of the Fourier transform of eq. (5) are shifted to twice the optical path difference (OPD) due to the decreased period of the oscillations, as illustrated in Fig. 1(b) with the solid curve being shifted to the dashed curve. The spacing between points of discrete sampling of the spectrum in ω-space, Δω, is fixed by the spectrometer, which fixes the depth range (both positive and negative OPD) to *z*_*S*_ = 2*πc*/Δω. It is therefore possible that peaks that were well below the Nyquist limit *z*_*N*_ = *z*_*S*_/2 before the multiplication are above after, reaching up to twice the Nyquist limit, as illustrated in Fig. 1(b). This effectively reduces the available depth range without aliasing (the Nyquist Sampling Theorem) by a factor of two compared to conventional SD-OCT^26^. In addition, the cross terms from multiplication between different cosines cause artefacts, which deteriorate the image quality^23,26,27,29^. For IC-SD-OCT to be relevant, the imaging depth must be restored, and the artefacts eliminated.

### Restoring the imaging depth using the analytical signal – ICA-SD-OCT

The coloured hatched areas in Fig. 1(b) indicate the IC-SD-OCT signal and aliased signal are trespassing into one another’s imaging range set by *z*_*N*_ (vertical dashed lines). To eliminate this aliasing problem we here, for the first time to our knowledge, propose to use the complex analytic interferograms in eq. (5), instead of the real-valued interferograms. The complex analytic signal *I*_*a*_ of a real signal I is computed by applying the Hilbert transform (HT),

6 $$\begin{array}{cc}{I}_{a}(\omega ) & \,=\,I(\omega )+i{\mathscr{H}}\{I(\omega )\},\\ {\mathscr{H}}\{\,f(\omega )\} & \,\equiv \,\frac{1}{\pi \omega }\otimes f(\omega )\end{array}$$

where ⊗ denotes convolution. The analytic signal is zero for negative OPDs by definition, and thus also for depths between *z*_*N*_ and *z*_*S*_ due to the repetition of the spectrum of discretely sampled signals, as illustrated in Fig. 1(c) (solid line). The components of the IC-SD-OCT interferogram that are deeper than the Nyquist depth (red part in Fig. 1(b)) are, when using the analytic signal, therefore fully distinguishable, i.e., aliasing is eliminated, as seen in Fig. 1(c) (dashed line). We term this the ICA scheme. As a result of any of the IC and ICA procedures, the density of points is doubled, but by using the ICA scheme, the imaging depth is maintained because all points are utilised and not only half.

Shirai *et al*. have theoretically investigated the application of IC-SD-OCT for multiple scattering samples in the special case where dispersion originates from only a dispersive element in the sample arm of the SD-OCT system^27^, i.e., neglecting the dispersion from the sample itself. Here we present an extended derivation that is based on a conventional SD-OCT set-up and takes the dispersion from the sample into account. We want to derive a theory for multiple scatterers because the IC- and ICA-SD-OCT procedures cause artefact to emerge due to the multiplication in eq. (5) creating cross terms. We therefore consider the simplest case with cross terms, which is with two scatterers, without loss of generality.

Using the analytic signal of eq. (4), Taylor expanding $\beta (\omega )=\sum _{j=0}^{\infty }\frac{{\beta }_{j}{\omega ^{\prime} }^{j}}{j!}={\beta }_{0}+{\beta }_{1}\omega ^{\prime} +\,{\beta }_{NL}^{(even)}+{\beta }_{NL}^{(odd)}$, with ${\beta }_{NL}^{(even)}=\sum _{i=1}^{\infty }\frac{{\beta }_{2i}{\omega }^{^{\prime} 2i}}{2i!}$, and ${\beta }_{NL}^{(even)}=\sum _{i=1}^{\infty }\frac{{\beta }_{2i+1}{\omega }^{^{\prime} 2i+1}}{(2i+1)!}$ containing, respectively, the even and odd non-linear terms of the dispersion, we find

7 $$\begin{array}{lrl}{I}_{n,a}({\omega }_{0}+\omega ^{\prime} ) & = & {r}_{1}^{2}+{r}_{2}^{2}+2{r}_{1}{r}_{2}{e}^{i{\rm{\Delta }}L[{\beta }_{0}+{\beta }_{1}\omega ^{\prime} +{\beta }_{NL}^{(even)}+{\beta }_{NL}^{(odd)}]}\\  &  & +2{r}_{1}{e}^{i[\frac{({\omega }_{0}+\omega ^{\prime} ){\rm{\Delta }}l}{c}+{L}_{1}({\beta }_{0}+{\beta }_{1}\omega ^{\prime} +{\beta }_{NL}^{(even)}+{\beta }_{NL}^{(odd)})]}\\  &  & +2{r}_{2}{e}^{i[\frac{({\omega }_{0}+\omega ^{\prime} ){\rm{\Delta }}l}{c}+{L}_{2}({\beta }_{0}+{\beta }_{1}\omega ^{\prime} +{\beta }_{NL}^{(even)}+{\beta }_{NL}^{(odd)})]}\end{array}$$

and from eq. (5)

8 $$\begin{array}{lrl}{I}_{ICA}(\omega ^{\prime} ,{\omega }_{0}) & = & {I}_{n,a}({\omega }_{0}+\omega ^{\prime} ){I}_{n,a}^{\ast }({\omega }_{0}-\omega ^{\prime} )\\  & = & {({r}_{1}^{2}+{r}_{2}^{2})}^{2}+4{r}_{1}^{2}{r}_{2}^{2}{e}^{i2({\beta }_{1}\omega ^{\prime} +{\beta }_{NL}^{(odd)}){\rm{\Delta }}L}+4{r}_{1}^{2}{e}^{i2([{\beta }_{1}\omega ^{\prime} +{\beta }_{NL}^{(odd)}]{L}_{1}+\frac{{\rm{\Delta }}l\omega ^{\prime} }{c})}\\  &  & +\,4{r}_{2}^{2}{e}^{i2([{\beta }_{1}\omega ^{\prime} +{\beta }_{NL}^{(odd)}]{L}_{2}+\frac{{\rm{\Delta }}l\omega ^{\prime} }{c})}+4{r}_{1}{r}_{2}({r}_{1}^{2}+{r}_{2}^{2}){e}^{i([{\beta }_{1}\omega ^{\prime} +{\beta }_{NL}^{(odd)}]{\rm{\Delta }}L+\frac{{\rm{\Delta }}l\omega ^{\prime} }{c})}\\  &  & \times \,\cos ([{\beta }_{0}+{\beta }_{NL}^{(even)}]{\rm{\Delta }}L)+4{r}_{1}({r}_{1}^{2}+{r}_{2}^{2}){e}^{i([{\beta }_{1}\omega ^{\prime} +{\beta }_{NL}^{(odd)}]{L}_{1}+\frac{{\rm{\Delta }}l\omega ^{\prime} }{c})}\\  &  & \times \,\cos (\frac{{\omega }_{0}{\rm{\Delta }}l}{c}+[{\beta }_{0}+{\beta }_{NL}^{(even)}]{L}_{1})+4{e}^{i([{\beta }_{1}\omega ^{\prime} +{\beta }_{NL}^{(odd)}]{L}_{2}+\frac{{\rm{\Delta }}l\omega ^{\prime} }{c})}\\  &  & \times \,[{r}_{2}({r}_{1}^{2}+{r}_{2}^{2})\cos (\frac{{\omega }_{0}{\rm{\Delta }}l}{c}+[{\beta }_{0}+{\beta }_{NL}^{(even)}]{L}_{2})\\  &  & +\,2{r}_{1}^{2}{r}_{2}\cos (\frac{{\omega }_{0}{\rm{\Delta }}l}{c}-[{\rm{\Delta }}L-{L}_{1}][{\beta }_{0}+{\beta }_{NL}^{(even)}])]\\  &  & +\,8{r}_{1}{r}_{2}^{2}{e}^{i([{\beta }_{1}\omega ^{\prime} +{\beta }_{NL}^{(odd)}][{L}_{2}+{\rm{\Delta }}L]+\frac{{\rm{\Delta }}l\omega ^{\prime} }{c})}\cos (\frac{{\omega }_{0}{\rm{\Delta }}l}{c}+[{\beta }_{0}+{\beta }_{NL}^{(even)}]{L}_{1})\\  &  & +\,8{r}_{1}{r}_{2}{e}^{i([{\beta }_{1}\omega ^{\prime} +{\beta }_{NL}^{(odd)}][{L}_{1}+{L}_{2}]+\frac{{\rm{\Delta }}l\omega ^{\prime} }{c})}\cos ([{\beta }_{0}+{\beta }_{NL}^{(even)}]{\rm{\Delta }}L)\end{array}$$

with * denoting complex conjugates. The four first terms in the second and third lines in eq. (8) are equivalent to the four terms from conventional OCT in eq. (7), but now positioned at twice the OPD and without any GVD from the dominant dispersion term β_2_ and all other even orders of dispersion. Contrary, the odd dispersion terms are not removed, and they are even enhanced by a factor of 2, but the dominating term, *2β*_3_, often has a much weaker effect than *β*_2_ has in conventional SD-OCT^30^. The remaining five terms are artefacts emerging from the cross terms of the multiplication in eq. (5), and they will be treated in the following section. In order to maintain the correct physical distance, the *z*-axis must be scaled by a factor $\frac{1}{2}$ as previously explained, and the point density is thus also increased by a factor of two, maintaining the imaging depth.

### Artefact reduction

As discussed above *I*_*n*_(*ω*_0_ − *ω*′) in eq. (5) can be obtained either experimentally or numerically by mirroring *I*_*n*_(*ω*_0_ + *ω*′). The ‘balanced detection’ experimental configuration has been shown to supress some of the artefacts in IC-SD-OCT^26,27^, i.e., some of the last 5 terms in eq. (8). It has also been shown that in the numerical configuration artefacts can also be removed, but only one at a time using a window function^26,29^. Very recently, Shirai showed that a numerical scheme can generically remove all artefacts in the dual-spectrometer configuration^28^. Here, we briefly go through the scheme, improve it slightly by introducing a weight function, both on basis of eq. (8), showing that the algorithm works equally well using a conventional SD-OCT setup, leaving the complex and expensive dual-spectrometer redundant.

**Figure 2:**
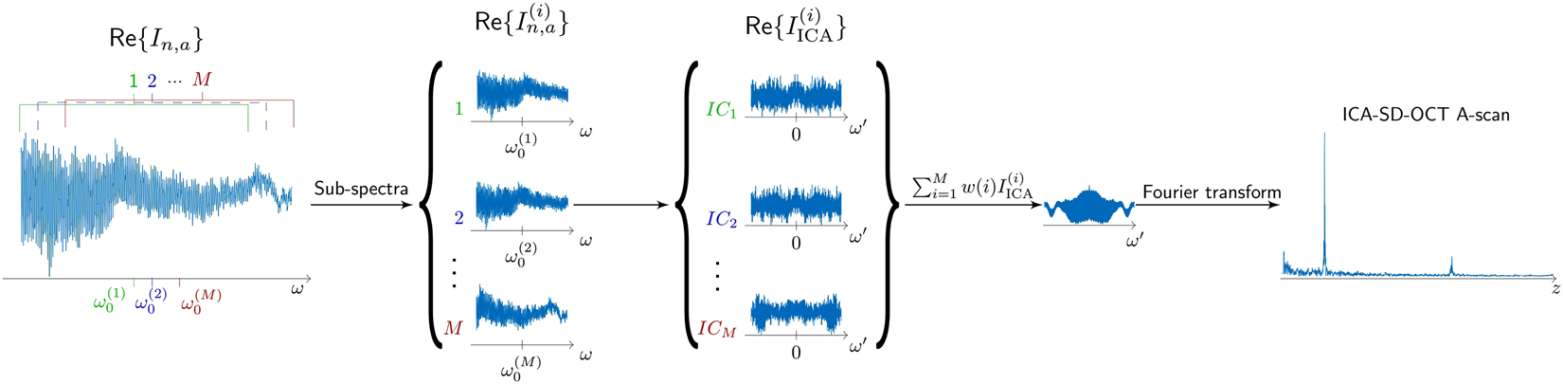
Flow chart illustrating the artefact reduction algorithm. The analytic signal of the spectrum is divided into M sub-spectra, which are treated individually to ICA spectra according to eq. (8). To obtain the final A-scan, the spectra are added with weights and Fourier transformed in *ω*′ before the square root is taken to return to go from squared reflectivity to reflectivity.

The five terms last artefact terms in eq. (8) all have a ~ cos *ω*_0_ dependence, either explicitly or implicitly through ${\beta }_{0}=\frac{{\omega }_{0}n({\omega }_{0})}{c}$. To reduce the artefacts, we employ a procedure based on varying the centre frequency. This helps identify the artefacts, as first noted by Banaszek *et al*.^23^. Varying the centre frequency of the source is challenging, and therefore a numerical procedure is implemented instead. A flowchart illustrating the process in seen in Fig. 2. The process works by numerically splitting the normalised, analytic spectrum *I*_*n*,*a*_ of length *N* into *M* sub-spectra of length *N* − *M* + *1*, whose centres are shifted 1 pixel relative to their neighbours’, as illustrated in panel 1 and 2 of Fig. 2. The first of the *M* spectra comprises the first *N* − *M* + 1 pixels of the full spectrum. The next sub-spectrum starts at pixel 2 of the full spectrum and so on, until sub-spectrum *M*, which is the last *N* − *M* + 1 pixels of the full spectrum. This procedure varies numerically the centre frequency at the cost of narrowing the spectrum by *M* − *1* pixels. The ICA-SD-OCT procedure of eq. (8) is then applied to all *M* sub-spectra independently, giving M ICA-SD-OCT sub-spectra, shown in panel 3 of Fig. 2. These spectra correspond to a span of *ω*_0_′s with a fixed *ω*′ axis, and because the artefacts oscillate in *ω*_0_, while the real OCT terms do not, the artefacts can be removed by averaging the *M* ICA sub-spectra. The ω_0_ span must be sufficiently large to ensure that the oscillations are averaged out effectively. Intuitively, this would require the span to cover at least one period of the oscillation, but that is, in fact not enough. Say the span of ω_0_’s covers a non-integer number of periods. The fraction of a period in the end of the span will then, when all the values are summed, leave a residual, such that the artefact will still be visible, and because the artefacts oscillate with different periods in ω_0_, it is not possible to choose the span to cover exactly an integer number of periods for all artefacts. This implies that the amount of periods needed are larger than one. However, applying a weighting function, *w*(*ω*_0_) for this final summation, shown in panel 4 of Fig. 2, greatly reduces the M-value required for sufficient artefact reduction. The weighting function weighs each sub-spectrum, such that the first has a lower weight than the second does, and the central sub-spectrum has the highest weight. This reduces the influence of the fractional periods in either end of the ω_0_-span, which in turn greatly reduces the residual, i.e., the artefact for a given *M*. As a result, the *M* value required to suppress the artefact to a given level is reduced when using the weights. As a rule of thumb when choosing *M*, we shall require at least 5 full oscillations of every artefact to ensure complete removal of all artefacts. The slowest oscillations are, in most cases, the ones with $\cos \,({\beta }_{0}{\rm{\Delta }}L)\approx \,\cos ({\omega }_{0}{\rm{\Delta }}L)$ in eq. (8), requiring

9 $$({\omega }_{0,max}-{\omega }_{0,min}){\rm{\Delta }}L\ge 5\times 2\pi \Rightarrow M=\frac{({\omega }_{0,max}-{\omega }_{0,min})}{\delta \omega }\ge 5\times \frac{2{z}_{S}}{{\rm{\Delta }}z},$$

where Δz is the smallest OPD between two reflectors. Here we used an *M*-point Hanning window as weights.

The ICA-SD-OCT A-scan is reached by performing a Fourier transform on the averaged ICA-SD-OCT spectrum. However, due to the spectral multiplication, all the reflection coefficients *r*_1,2_ are also squared, and the resulting depth scan is thus a profile of the squared reflectivity instead of just the reflectivity. To re-obtain the OCT reflectivity profile (first order in reflectivity) the square root of the depth scans are evaluated.

The implication of the narrowing of the spectrum by *M* − *1* pixels depends on the hardware employed. When using a source with a Gaussian-like spectrum, the narrowing will not matter much. The Gaussian shape means that the lost pixels near the edges have little amplitude. However, in this study we use an ultra-broadband supercontinuum source, which ensures the interference pattern covers the entire range of the spectrometer (see Methods). In this case, narrowing the spectrum will consequently also deteriorate the axial resolution with a factor of *N*/(*N* − *M* + *1*), meaning that an optimal *M*-value can be determined as a trade-off between the quality of the artefact reduction and the deterioration of the axial resolution.

Finally, the processing time must be discussed. In addition to conventional SD-OCT processing, ICA-SD-OCT requires a Hilbert transform on the full spectrum, *M* splits of the full spectrum, *M* × (*N* − *M* + 1) floating point multiplications to compute the *M* ICA sub-spectra, and as many additions for the averaging. For *M* ≪ *N*, the number of operations are proportional to *M* × *N*, i.e. the processing time scales linearly with *M*. Typical *M*-values are in the range 100–200 (see Results), and so, the time to process an IC-SD-OCT image is typically hundreds of times slower than conventional OCT making it unsuited for real-time applications. However, the processing of each A-scan is independent, and thus a heavily parallelized GPU-implementation could make a real-time imaging available.

### OCT axial resolution in IC-OCT

In the literature IC-OCT, both TD and SD, is generally claimed to have a $\sqrt{2}$ better axial resolution than conventional OCT^21,23,25–27,29,31,32^. However, we find this to be misleading because it originates from not defining the axial resolution from the same signal, i.e., conventional OCT defines it from the reflection profile, whereas IC-OCT defines it from the squared reflection profile. In IC-SD-OCT for example, the intensity spectrum is after the Michelson interferometer mirrored and combined with itself, whereas in for example chirped-pulse IC-TD-OCT two oppositely chirped pulses are combined. In other words, IC-OCT in general exploits fourth-order correlations in that it combines two intensity spectra, i.e., four complex field spectra, whereas conventional OCT exploits second-order correlations.

However, if the original signal that is about to be squared in IC-OCT, cannot resolve two closely spaced reflectors but shows them as a single peak, then the squared signal will also only show a single peak. Thus, if the resolution was not defined as the FWHM of the A-scan of a single mirror, but as the distance between reflectors the system is able to resolve, then there would be no improvement in resolution with IC-OCT.

We would like to note that a “true” resolution improvement of $\sqrt{2}$ compared to standard (classical) OCT is found in so-called quantum OCT, which by nature requires two detectors and therefore inherently is IC-OCT, as demonstrated in^17,32^. This stems from the spectral entanglement shared between two photons. One photon travels the path of the reference arm and the other the arm of the sample. The two photons are subsequently mixed on a beam splitter (as in conventional OCT), after which a coincidence event is recorded varying the relative time delay (scanning the reference arm length similar to the procedure employed in TD-OCT), also known as the Hong-Ou-Mandel interferometer^33^. The A-scan so obtained is in fact assimilated to a coincidence curve. For equal paths lengths a dip in the coincidence curve enabled by the unique temporal and spectral correlations between the two photons is observed. Due to the spectral entanglement between the two photons, the FWHM of this curve (assuming a Gaussian shape) is indeed a factor of $\sqrt{2}$ smaller than the FWHM of the A-scan of a mirror in conventional (classical) OCT, even when comparing the same orders of field reflectivity. This resolution improvement can only be explained by non-classical correlations between the two photons^34,35^.

### Numerical simulations

**Figure 3:**
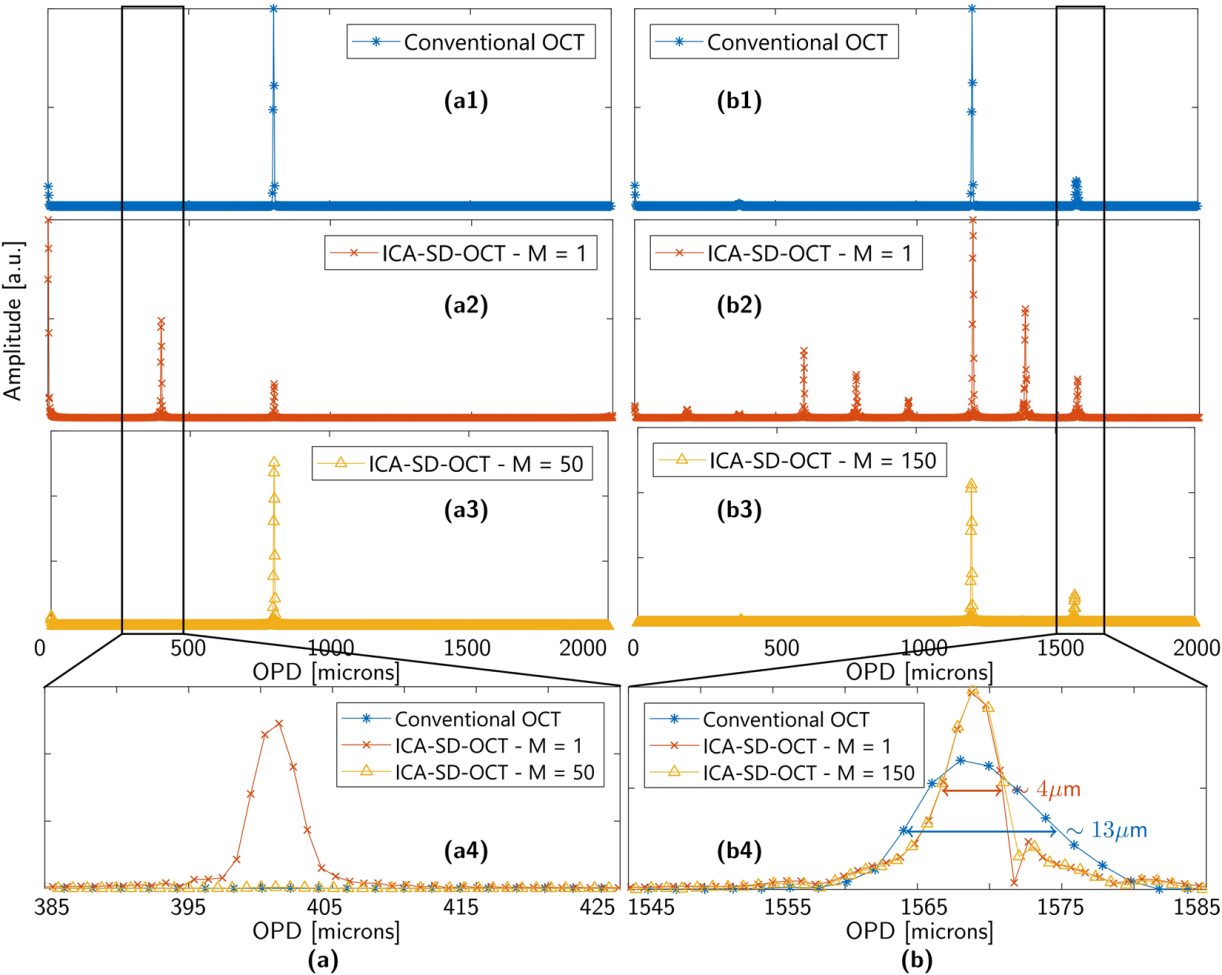
Simulated data to illustrate the difference between conventional OCT and IC-OCT for a single reflector (**a**) and two reflectors (**b**). (a/b1–a/b3) shows conventional OCT, ICA-SD-OCT without the windowing procedure, and ICA-SD-OCT with the windowing procedure applied, respectively. (a4) and (b4) show zoom-ins of the artefact and the dispersion compensated peaks, respectively. The simulations are done with 2048 points spaced between 1070 nm and 1470 nm evenly in *k*-space, giving an imaging depth of 2 mm and a pixel distance of 1.97 microns. The source was simulated as a Gaussian spectrum with a central wavelength of 1300 nm and a FWHM of 230 nm.

We carried out a proof of principle simulation to test our ICA-SD-OCT approach. Figure 3(a1–a4) and (b1–b4) show the ICA-SD-OCT procedure applied to simulated data with one and two reflectors, respectively. For a single reflector conventional OCT is shown in Fig. 3(a1), ICA-SD-OCT without artefact reduction (M = 1) is shown in Fig. 3(a2), and ICA-SD-OCT with *M* = 50 artefact reduction is shown in Fig. 3(a3). An artefact emerging from the cross term between the single reflector and the DC term is seen at ~400 microns in Fig. 3(a2), which is clearly suppressed by the *M* = 50 artefact reduction, as seen in Fig. 3(a3) and the zoom in Fig. 3(a4). $M=50\ge 5\times \frac{2{z}_{S}}{800\,\mu \,m}$ satisfies the criterion that the artefact (term 6 in eq. (8)) oscillate 5 periods in the ω_0_ span. Figure 3(b) shows the result of a simulation of OCT imaging of a 100 micron thick silicon plate. The refractive index of silicon used for the simulation is the experimental data provided in^36^ and then interpolated to fit our spectral pixels by a standard piecewise cubic Hermite interpolating polynomial (PCHIP) routine. Figure 3(b1–b3) shows the same as 3(a1–a3), but for two reflectors. In this case Fig. 3(b2) shows nine peaks including the DC term (1 DC term, 2 reflectors, 1 cross term from conventional OCT, and 5 ICA-SD-OCT artefacts), which corresponds directly to the nine terms in eq. (8).

Figure 3(b1) constitutes the baseline for what is possible to achieve with ICA-SD-OCT in terms of artefact reduction. The two reflectors at 1200 microns and 1550 microns, stemming from the silicon plate (*n* = *3.5*), and the cross term at 350 microns are the three peaks that should be left after the ICA-SD-OCT windowing procedure. Figure 3(b3), which shows the result for ICA-SD-OCT with M = 150 artefact reduction, demonstrates that M = 150 is enough to suppress the ICA-SD-OCT artefacts and recover the 3 peaks from Fig. 3(b1), as expected from the limit in eq. (9), which gives *M* = 115.

Figure 3(b4) shows a zoom-in of the back face of the silicon plate, which for the case of conventional OCT is broadened by GVD, but for ICA-SD-OCT, both with and without artefact reduction is restored to its GVD-free width. The slight increase in FWHM observed for M = 150 is negligible, but for larger M values the broadening becomes more severe. A compromise between the artefact reduction and the resolution deterioration thus has to be established.

## Methods

**Figure 4:**
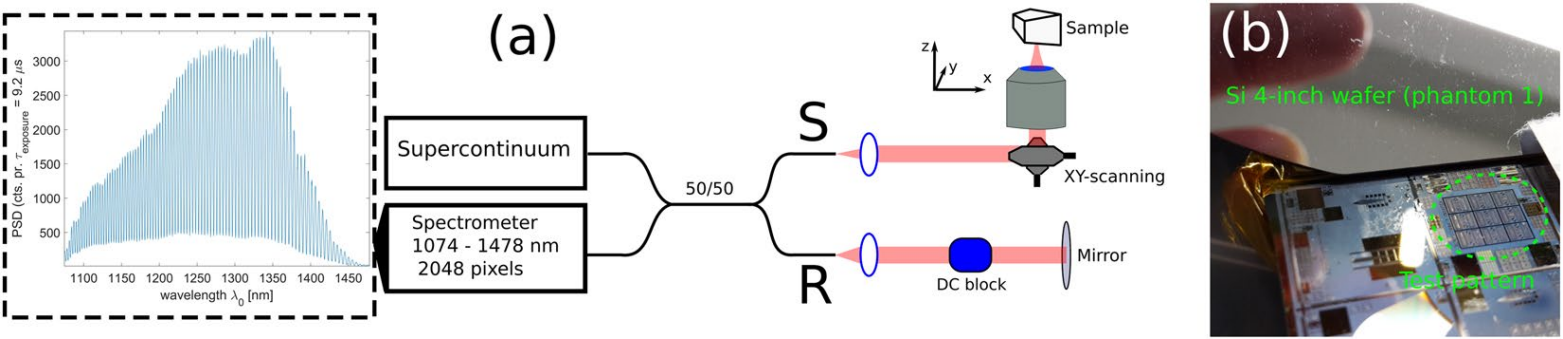
(**a**) Sketch of the experimental SD-OCT set-up. The broadband NIR light is split evenly into a reference arm (R) and a sample arm (S). The interferometric signal is detected in the fourth arm of the coupler, and an example of an interferogram is displayed. (**b**) Shows a photograph of one of the phantoms imaged.

For imaging we used the conventional UHR SD-OCT system sketched in Fig. 4, which was recently used in clinical skin studies on healthy patients to show that UHR SD-OCT provides superior resolution sufficient to accurately delineate the dermal-epidermal junction^37^ and how nano-particles could improve the contrast in the OCT images^38^. As optical source, we used a 320 MHz superK Extreme EXR-9 OCT system (NKT Photonics A/S) with a long-pass filter selecting light in the range 1000–1750 nm. This high repetition rate supercontinuum source is especially suited for SD-OCT^39^. A 50/50 fibre coupler customized for 1300 nm (Goosch and Housego, Netherlands) wavelength, served as the beam splitter and standard achromatic lenses collimated the light in each output arm. In the sample arm, galvanometer scanners were deployed for scanning of the sample through a microscope objective (LSM02, Thorlabs, UK). In the reference arm, a block of glass was placed before the mirror for approximate hardware DC. Interferograms were recorded with a 1300 nm spectrometer C-1070-1470-GL2KL (Wasatch, USA) providing a ~400 nm bandwidth and operating at a line rate of 76 kHz.

The spectrometer non-linearity between wavenumber and pixel number is eliminated by re-sampling using two reference interferograms collected with a mirror placed at two different axial positions, as in^11^. This technique can also be used for standard single-reflector DC, which we will compare with IC-SD-OCT all-depth multi-reflector DC in the following. With the standard DC, an axial resolution of 3–5 µm (FWHM of Gaussian fit) over the entire 2 mm image range was measured (using a mirror as sample). Laterally we found our system to be able to distinguish features down to 6 *μ*m (USAF target 1951 phantom). For a power of 2.4 mW on the sample, the sensitivity is 89 dB. All interferograms are filtered with a 1300 nm Tukey window in *ω*′ with bandwidth 300 nm to smoothen the image. All A-scans and B-scans presented are single shot images with no temporal averaging applied.

### Data availability

The datasets generated and/or analysed in the current study are available from the corresponding author on reasonable request

## Results

To verify the theory and the results of the simulation, we imaged two phantoms. Standard DC, as described in the methods section, was applied only where mentioned explicitly. Phantom 1 is a polished silicon wafer of thickness 255 microns. The GVD of crystalline silicon is estimated to be 1100 ± 100 fs^2^/mm^36^, which is sufficient to cause significant broadening of the interface corresponding to the bottom surface of the wafer. Assuming a Gaussian spectrum, the relative broadening factor, *p*, due to GVD is calculated as:

10 $$p=\sqrt{1+{L}^{2}{\beta }_{2}^{2}{(\frac{\pi c{\rm{\Delta }}\lambda }{\sqrt{2{\rm{l}}{\rm{n}}2}{\lambda }_{c}^{2}})}^{4}}$$

Here *L* is the physical axial position relative to the surface of the sample, in this case 255 microns. *β*_2_ = ∂^2^*β*/∂*ω*^2^ is the GVD parameter, c is the speed of light in vacuum, and Δ*λ* and *λ*_*c*_ are the FWHM and centre wavelength, respectively. From the estimated GVD parameter, we expect a relative broadening of the bottom surface by a factor of ~4.1 ± 0.3.

**Figure 5:**
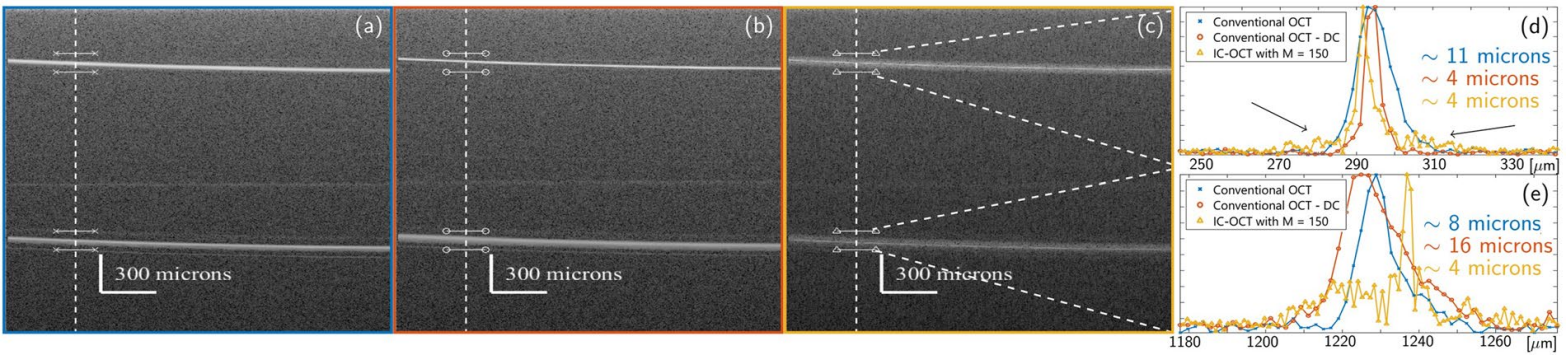
B-scan of a polished silicon wafer with (**a**) no DC, (**b**) global compensation of system dispersion, and (**c**) ICA-SD-OCT image with M = 150. (**d**) shows pieces of the A-scan along the white dashed line in (**a**–**c**). All images are single shot and filtered with a Tukey window.

Cross sectional images, B-scans, of the silicon wafer are shown in Fig. 5, with 5(a) being the image collected without any DC, 5(b) the image with conventional DC of the top interface, and 5(c) the ICA-SD-OCT image. Figure 5(d) shows the profile along the vertical dashed lines between the short horizontal, solid lines. The image with no DC in Fig. 5(a) shows the two surfaces having approximately the same thickness of ~10 microns despite the highly dispersive sample. The top interface is broadened due to the dispersion in the set-up, while the bottom interface is broadened by the combined effect of the dispersion in the set-up and in the sample. As the set-up dispersion and sample dispersion have different signs, the accumulated dispersion for the bottom interface is in magnitude smaller than the set-up dispersion, and therefore the bottom interface is thinner in the image than the top interface (but one is not always that lucky!). The image in Fig. 5(b) displays a narrow top interface with a FWHM of 4 microns and a bottom interface with a thickness that has increased by a factor of approximately 4 to 16 microns, as expected from eq. (10). The extra broadening of the bottom surface is due to the set-up dispersion having been cancelled, and it highlights the major drawback of conventional DC: Not all depths can simultaneously achieve the theoretical dispersion-free axial resolution. As shown in Fig. 5(c) the ICA-SD-OCT method allows thinning of all interfaces to about 4 microns simultaneously, irrespective of depth, by intrinsic cancellation of all even order GVD. The ICA-SD-OCT image is created with M = 150 sub-spectra, which allows to obtain significant reduction of the artefacts originating from cross terms between two scatterers seen in Figure 4(a2) and (b2), with no trace of these artefacts even on a logarithmic scale. We note however that the ICA-SD-OCT procedure introduces a weak set of artefacts in the background of each reflector peak, seen as the blur around both surfaces in Fig. 5(c), and as side lobes in Fig. 5(d), as marked by the black arrows. These noise side lobes stem from the cross term between a scatterer and the background noise, which was not included in the theoretical derivation or the numerical simulations. The width of these side lobes, that appear around every reflector peak, decreases with an increasing *M* number. However, since the side lobes are a direct consequence of the noise in the system, the side lobes can also be reduced by employing a low-noise source. In a simple phantom as imaged here, the side lobes do not obscure the signal, but in a complex biological sample, this not generally the case. The optimal *M* values does thus also require sufficiently reduced side lobes, where the level deemed sufficient will depend on the sample.

**Figure 6:**
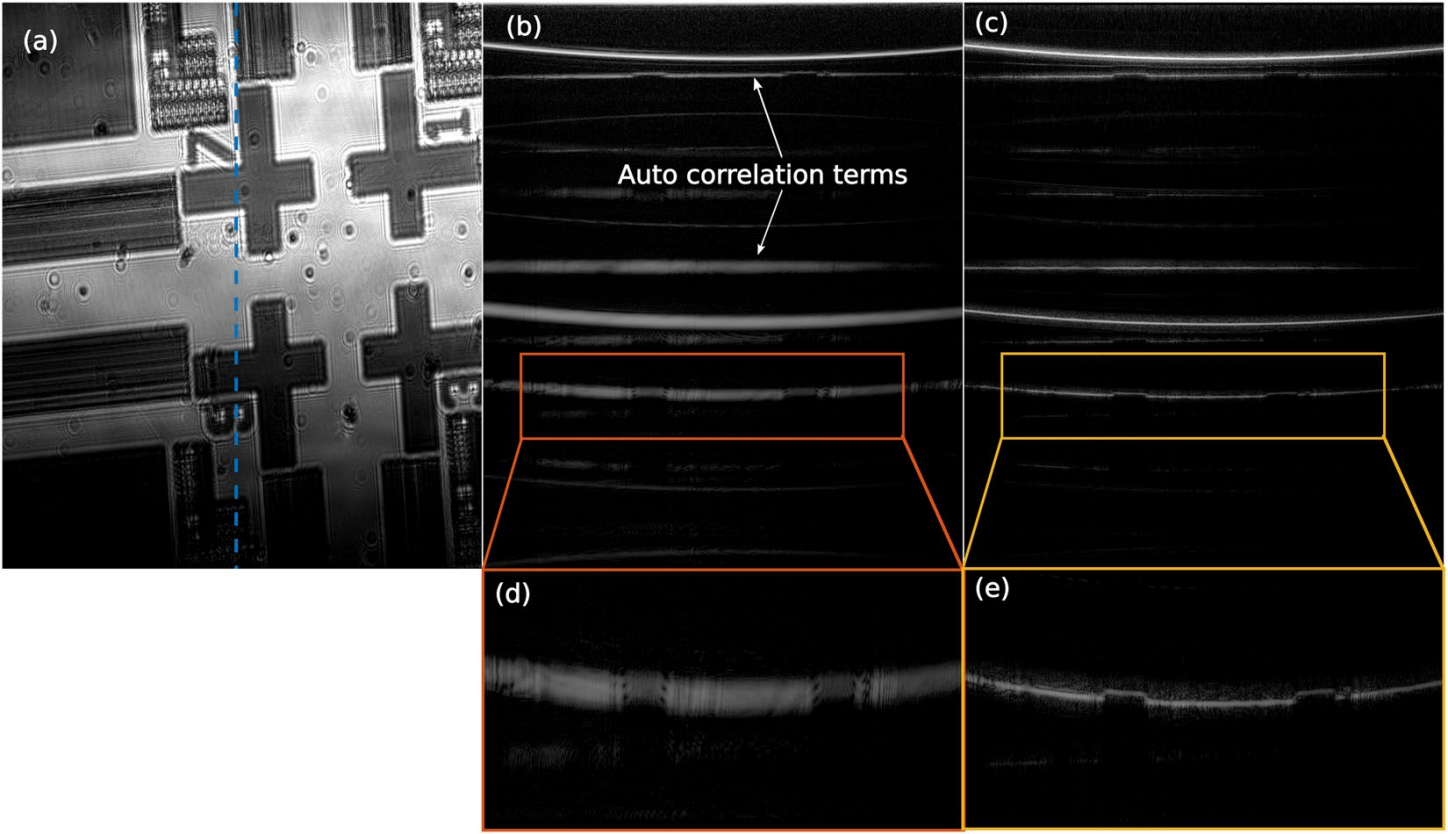
OCT images of a silicon wafer with surface structure placed beneath an ordinary silicon wafer. (**a**) Shows a top view of the surface structure, (**b**) shows a B-scan along the dashed blue line with conventional DC applied, and (**c**) shows the same B-scan using ICA-SD-OCT. (**d**) and (**e**) show a zoom of the structure from (**b**) and (**c**), respectively, highlighting the superior level of detail of ICA-SD-OCT.

To further evaluate the performance of the ICA-SD-OCT procedure, we created phantom 2 by placing a silicon wafer with a surface structure below phantom 1. The resulting images are seen in Fig. 6 with (a) showing a top view OCT image of the structured surface, and (b) and (c) the B-scan along the blue line in (a) with conventional DC and ICA-SD-OCT respectively. (d) and (e) show zoom-ins of the structured surface of (b) and (c), respectively. This phantom imitates a sample with several interfaces and small scale features deep inside it. From Fig. 6(b,d) we see how an SD-OCT system with conventional DC is not able to clearly visualise the fine details of the structure on the bottom wafer. In contrast, in Fig. 6(c,e), the ICA-SD-OCT image with M = 200 artefact reduction provides such a good all-depth DC that the structure is clearly visible. The blurry parts in Fig. 6(d) are clearly seen in Fig. 6(e) to be elevated slightly relative to the rest of the wafer, a detail not visible in Fig. 6(d). From Fig. 6(c) we can appreciate that ICA-SD-OCT restore the two surfaces of the top wafer and the top of the bottom wafer to their dispersion free widths.

## Summary and Conclusions

In summary, we have theoretically and experimentally demonstrated a new ICA-SD-OCT procedure that allows all-depth DC of all even order GVD. We show that we numerically can eliminate the GVD due to the sample, irrespective of the scattering depth, allowing us to maintain the theoretical axial resolution at all depths, and this using a conventional SD-OCT set-up with only a single spectrometer. Furthermore, our new numerical procedure maintains the axial range, which was not possible before, as well as generically removes all artefacts emerging from the multiplication of two interferograms. Numerical simulations were performed using a single and dual layer sample to investigate the dispersion compensating abilities and artefact reduction of the ICA-SD-OCT procedure. These simulations demonstrated tolerance to GVD from the sample as well as excellent reduction of the artefacts. Two phantoms were imaged experimentally, a single polished silicon wafer, and the same polished silicon wafer with another structured silicon wafer placed underneath it. We demonstrated how a conventional SD-OCT system with conventional single-reflector DC, showed a severely broadened bottom surface due to sample dispersion and was not able to clearly image the surface structure of the bottom wafer. In contrast, our experimental results showed how ICA-SD-OCT processing can compensate dispersion in-depth and image the bottom small features 260 microns into the phantom and re-establish a 4-micron resolution for both top and bottom surfaces.

In the scope of increasingly applied supercontinuum sources, multi-layer DC becomes gradually more relevant as the optical bandwidth is increased to improve the axial resolution of OCT systems, and to this end, ICA-SD-OCT is ideal because the dispersion is automatically and intrinsically removed. Only the number of windowed spectra M around the central frequency *ω*_0_, needs to be chosen, but this can become a constant for a given sample, such that after initial tuning of M, imaging can proceed as with conventional OCT. We expect that this procedure will be particularly useful for non-destructive testing and metrology, where highly dispersive samples are common.

Following the recipe of ICA-SD-OCT, all present SD-OCT systems can operate as ICA-SD-OCT systems. A future necessary step towards advancing the applicability of ICA-SD-OCT is to investigate the noise properties further and sensitivity, which we have not included in this study.
